# Antipyrin a Hæmostatic

**Published:** 1892-07

**Authors:** 


### ANTIPYRIN A HÆMOSTATIC.

Dr. F. C. Ford, of Nacodoches, Texas, Medical Director of Camp Mabry, Division of Texas Volunteer Guard, State Troops, now in annual encampment at Austin, says that antipyrin is a prompt styptic, and can be relied upon to arrest hemorrhage when other remedies fail. He made the discovery recently; being called upon to remove a small vascular tumor in the mouth, an apparently insignificant matter, he had difficulty which he did not anticipate in stopping the blood. He touched it first with a point of nitrate of silver and it had no effect; he then applied antipyrin, in powder, with the result as stated, of immediately arresting the flow.

The Doctor does not know what prompted him to do it, as he had never heard it recommended; nor was it suggested by the knowledge that antipyrin possesses any astringent properties; it is just one of those clinical facts which one cannot explain, and is, therefore, empirical. Can any of the JOURNAL'S readers explain its action? or have any of them had a similar experience?

Dr. Ford further states that antipyrin can be relied upon in hemorrhage after extraction of a tooth; he has not tried it in other cases. Should experience confirm the Doctor's observation, and establish the fact that it was not a co-incidence, but that the drug is really a hemostatic, the discovery is of great practical value. All know the difficulty sometimes encountered in arresting apparently trifling hemorrhage. In epistaxis, for instance, if it can obviate plugging it will be a most valuable acquisition to the surgeons' resources. The solution of per-chloride of iron, the tincture of iron, and other preparations of the kind are often objectionable, while being, next to ligature, his principal resource. They leave an ugly sediment or deposit upon the wound which has to be removed, and are sometimes contra-indicated by their irritant nature, Antipyrin will make a much more cleanly dressing, to say the least. Make a note of this.

---

THE "MILK IN THE COCOANUT," AND OTHER THINGS.—Since Dr. West's letter was in print we have received a letter from an old member who had paid his dues for Dr. West's first year complaining that his name did not appear in that list of members which good Dr. West says he worked so long and hard to "perfect;" complaining, also, that when he appealed to Dr. West and protested, the doctor wrote him "it was not *his* fault." Whose, then, was it? This same letter says that Dr. Larendon, the Treasurer, is pressing him for this year's pay, $5 (which the writer didn't pay, because his name had been omitted, notwithstanding he paid in advance), stating that "every dollar that can be raised *is needed in order to enable the Committee to get out the Transactions.*" The writer of this letter is the venerable and beloved Dr. Paulus. How does this comport with Dr. West's boasts?

We tender the doctor an apology for offering to assist him in proof-reading. It was kindly meant, intended as a courtesy. Having had eight years experience in editing Transactions, and ten in proof-reading, and the doctor having *invited our assistance*, we offered it,believing the courtesy, at least,would be appreciated; but as he denounced it as "a cool proposition," and suspected us of designs of supplanting him—and lets the world know that *He* is Secretary and that we can't make any figure-head of him, —we beg his pardon; we really *didn't* know the doctor *knew all about it!*

---

DR. GEORGE DOCK, of Ann Arbor, Mich., whom our readers will remember as the professor of Bacteriology, Pathology and Microscopy in the Texas Medical College at Galveston before it was absorbed by the University of Texas and became the Medical Department of that University, was married in Galveston on the 6th of July, inst., to Miss Laura McLemore, daughter of Col. J. W. McLemore, a prominent lawyer of that city. In addition to his other "ologies" he will now, doubtless, sing (the) Dock's-ology.

